# ddRAD Sequencing Identifies Pesticide Resistance-Related Loci and Reveals New Insights into Genetic Structure of *Bactericera cockerelli* as a Plant Pathogen Vector

**DOI:** 10.3390/insects13030257

**Published:** 2022-03-04

**Authors:** Mahnaz Kiani, Zhen Fu, Adrianna Szczepaniec

**Affiliations:** 1 Department of Entomology, Texas A&M AgriLife Research, 6500 Amarillo Blvd. W, Amarillo, TX 79106, USA; 2 U.S. Department of Agriculture, Agricultural Research Service, Beltsville, MD 20705, USA; 3 Department of Entomology, Texas A&M University, College Station, TX 77840, USA; daisy.fu@vai.org; 4 Bioinformatics and Biostatistics Core, Van Andel Institute, Grand Rapids, MI 49503, USA; 5 Department of Agricultural Biology, Colorado State University, Fort Collins, CO 80523, USA; a.szczepaniec@colostate.edu

**Keywords:** SNP discovery, potato psyllid, genetic population structure, insecticide resistance

## Abstract

**Simple Summary:**

Insect vectors of plant diseases and insecticide resistance pose the greatest challenge to sustainable crop production in food security. In this study, we explored genetic mechanisms responsible for resistance to a common insecticide group, neonicotinoids, in a key insect vector of potato diseases—potato psyllids. These small insects with piercing-sucking mouthparts are common in potato and can transmit a bacterium that causes zebra chip disease. Zebra chip has had a devastating impact on potato producers and has contributed to a highly regimented and intense use of insecticides to suppress potato psyllids as soon as they are detected in a field, commonly using neonicotinoids. Widespread resistance to these insecticides is now evident in potato psyllid populations. Using susceptible and resistant psyllid populations, we sequenced portions of their genomes to elucidate genes involved in the evolution of resistance to neonicotinoid insecticides. We found several genes that are likely to be responsible for insecticide resistance, and these should be explored in further research. We also discovered that a method commonly used to separate potato psyllids into distinct groups based on their geographic origin does not adequately represent their genetic population structure and should be used in conjunction with other genetic techniques.

**Abstract:**

(1) Background: Many hemipteran insects transmit plant pathogens that cause devastating crop diseases, while pest management frequently relies primarily on insecticide applications. These intense insecticide applications lead to the development of insecticide resistance, as was the case for potato psyllid, *Bactericera cockerelli* (Hemiptera: Triozidae), a vector of *Candidatus* Liberibacter solanacearum, which causes zebra chip disease in potato. (2) Methods: Here, we use double-digest restriction site-associated DNA (ddRAD) to genotype eight psyllid populations (one susceptible and seven resistant to neonicotinoid insecticides). (3) Results: Association tests identified over 400 loci that were strongly segregated between susceptible and resistant populations. Several loci were located within genes involved in insecticide resistance, gene regulation, fertility, and development. Moreover, we explored the genetic structure of these eight populations and discovered that routinely utilized haplotyping was not an accurate predictor of population structure. Pairwise comparisons of the fixation index (*F*_ST_) of populations of the same haplotype were not different from pairwise *F*_ST_ of populations that belonged to different haplotypes. (4) Conclusions: Our findings suggest that neonicotinoid insecticide resistance has a genetic basis, most likely as a result of similar selection pressure. Furthermore, our results imply that using a single maternally inherited gene marker to designate genetic lineages for potato psyllids should be re-evaluated.

## 1. Introduction

Many insects in the order Hemiptera, such as aphids, psyllids, leafhoppers, and whiteflies, are highly injurious to crops due to their wide host range and rapid reproduction. Their injury is more severe when these insects transmit plant pathogens—bacteria, viruses, phytoplasmas, and fungi [1,2]. Management of these insect vectors is challenging, and chemical control aimed to suppress the insects remains the most widely implemented approach [1,3,4,5]. The widespread application of insecticides can disrupt ecosystem services that are dependent on natural enemies of pests, contribute to outbreaks of other primary arthropod pests, and cause outbreaks of secondary diseases transmitted by insects [6,7,8]. Further, insecticide-resistant populations have been reported in many crop systems globally [9,10,11,12,13,14], reducing the efficacy of pest management programs.

The potato psyllid, *Bactericera cockerelli* (Šulc) (Hemiptera: Triozidae), is a vector of pathogens that are casual agents of several diseases, including “psyllid yellows” (PY) [15], “zebra chip” (ZC) [16], and “vein greening disease” [17]. Among these, ZC is particularly injurious to potato, resulting in significant losses to producers and contributing to extremely intense pesticide applications to suppress potato psyllids. Zebra chip disease is caused by a bacterial pathogen, *Candidatus* Liberibacter solanacearum (Lso) [18,19], and has been responsible for extensive economic losses in most potato production regions of the US. *Bactericera cockerelli* is thought to be native to Central and North America and its current distribution ranges from southern Canada to Central America, New Zealand, and Australia [20,21]. Four different haplotypes of the potato psyllid have been identified based on the variation of Cytochrome c oxidase subunit I (COI) gene, Central, Western, Northwestern, and Southwestern, and the distribution of these haplotypes in the US roughly corresponds to geographical regions where the insects are prevalent [22,23]. Notably, the haplotype does not appear to affect the efficiency of Lso transmission or the likelihood of infection with the pathogen [24]. 

Owing to the lack of effective control measures for any of the pathogens transmitted by the potato psyllid, management is focused on the suppression of the psyllids and relies solely on pesticides [25,26]. The most frequently used systemic insecticides are neonicotinoids, which are commonly applied in-furrow during planting, as well as foliar sprays mid-season. Imidacloprid and thiamethoxam are the two most predominant neonicotinoids that are applied to soil or seed tubers [27,28]. Both insecticides are systemic and act as insect neurotoxins [29,30]. There is now widespread evidence that psyllids are resistant to these two commonly used neonicotinoid insecticides [14,31,32]. In fact, in our previous study, we demonstrated high resistance ratios in populations of the psyllids collected across potato-producing regions of Texas, New Mexico, and Colorado compared with a susceptible colony collected from the Lower Rio Grande Valley in Texas in 2006, prior to the intense use of neonicotinoids [14].

While the incidence and severity of the potato psyllid resistance to neonicotinoid insecticides have been documented in several separate studies, the mechanisms of this resistance are not known. Generally, hypotheses about molecular mechanisms of insecticide resistance are centered around targeted site mutations, which result in target insensitivity and increased expression of detoxification genes [9,11,12,13,33,34,35]. In previous RNA-seq or genome-wide association studies, a few genes associated with insecticide resistance have been identified [35,36,37,38]. However, genetic responses to insecticides are heterogeneous, as insect pests adapt to different and changing environments [38,39], and many other mechanisms that are outside these well-studied molecular mechanisms could contribute to insecticide resistance.

Here, we used genome-wide single nucleotide polymorphisms (SNPs) to investigate the genetic basis of resistance to neonicotinoid insecticides in resistant and susceptible potato psyllid populations collected across the potato-growing regions of the Southwestern US. We were interested in elucidating the genetic structure of the potato psyllid population and exploring the hypothesis that the selective pressure of the neonicotinoid insecticide on the potato psyllid population could result in heritable SNPs. In addition, we attempted to explore the genetic structure of the psyllid populations that are designated as Central and Western haplotypes. Given that the rapid evolution of insecticide resistance is a major challenge for sustainable agriculture, understanding the genetic resistance mechanisms is important for DNA-based resistance monitoring in field populations.

## 2. Materials and Methods

### 2.1. Psyllid Collections and Colony Maintenance

Psyllid colonies were established from adult psyllids collected during 2013, 2015, and 2016, except for the Texas (TX) LRGV 2006 colony, which was originally collected in 2006 from the Lower Rio Grande Valley of Texas and was previously shown to be susceptible to neonicotinoids [14,28]. Potato psyllid colonies were maintained in the greenhouse complex at Texas A&M AgriLife Research Plant Stress Laboratory in Bushland, TX. Eight potato psyllid populations that were designated to two haplotypes (‘Western’ and ‘Central’) were included in this work (Table 1). All potato psyllids were maintained on tomato plants (*Solanum lycopersicum* L., variety Lance) in 60 × 60 × 60 cm insect-proof mesh cages (MegaView Science Education Services Co., Taipei, Taiwan) in the greenhouse with mean temperatures of 21 ± 3 °C (night) to 30 ± 3 °C (day) and 16:8 L:D photoperiod.

### 2.2. Sample Collection and DNA Extraction

Six biological samples (replicates) consisting of five adult psyllids pooled together were used from each of the eight populations (Table 1). DNA was extracted using Puregene Core Kit A (Qiagen, Valencia, CA, USA). DNA quality and quantity were assessed using the NanoVue Plus spectrophotometer (GE, Healthcare, Piscataway, NJ, USA) and an Agilent 2100 Bioanalyzer (Agilent Technologies, Santa Clara, CA, USA) prior to ddRAD library preparation. The ddRAD-tag libraries were prepared from the genomic DNA of 48 samples using the restriction enzymes Spel and Mbol. The libraries were sequenced on one lane of the Illumina NovaSeq (Illumina, Inc., San Diego, CA, USA) with the 150 bp paired-end mode. The ddRAD library preparation, sequencing, and data demultiplexing were conducted by Texas AgriLife Research Genomics and Bioinformatics Center (College Station, TX, USA).

### 2.3. Data Analysis

#### 2.3.1. SNP Calling

We took a de novo approach using Stacks v2.41 [40] because there was no reference genome available for potato psyllid. Quality filtering was performed with the process_radtags module in Stacks with default parameters. Putative orthologous tags (Stacks) per biological sample were assembled using ustacks with a minimum depth of coverage required to create a stack (m) of three and a maximum nucleotide mismatch (M) of three. Catalogs of loci were assembled using cstacks; the number of mismatches allowed between sample loci when generating the catalogs (n) was six. Matches of individual RAD loci to the catalog were searched using sstacks. Finally, we used the populations module of Stacks to filter and export the genotype calls and computed several common population statistics. The following filters were used for SNP calling: loci should be present in at least 85% of the samples and also in six populations with minimum allele frequency (MAF) > 0.05, minimum minor allele count (minmac) ten, maximum observed heterozygosity 0.7, and *p*-value cutoff 0.05 for keeping an *F*_ST_ measurement. To obtain a putatively neutral SNP data set for genetic structure analysis, an “unlinked” data set was generated with a single SNP per locus (the first SNP per locus) to reduce the effects of linkage. We then used BayeScan v2.01 [41], which estimates the posterior probability of SNPs under selection. We removed the outlier SNPs from the original vcf file using VCFtools [42] and used this filtered data set (neutral SNPs) for genetic structure analysis.

#### 2.3.2. Genetic Basis of Insecticide Resistance Using Association Test and Annotation of De Novo Assembled Contigs

To identify loci that were strongly segregated between the insecticide susceptible (TX LRGV) and resistant populations (the remaining seven populations), we performed association analysis with the entire data set containing 12,681 variant sites using PLINK [43] (v1.9), and the *p*-value cutoff was set to 1 × 10^−14^.

Gene annotations were obtained for all specific RAD loci/contigs that contained significant SNPs in the association test using the Blast2GO annotation methodology embedded in OmicsBox (v 1.2.4; BioBam Bioinformatics S.L., Valencia, Spain). Sequences were queried against the Hemiptera database under “insects” (Taxid:6960), using OmicsBox with an E-value threshold of 1 × 10^−4^.

#### 2.3.3. Genetic Structure and Variation

We deployed three complementary approaches to model population genetic structure without a priori population assignment. First, we conducted principal component analysis (PCA) implemented in the PLINK, using neutral SNPs marker set. Secondly, we used Structure software (v 2.3.4 [44]) to explore the population genetic structure based on our neutral SNP marker set. The optimal number of populations present within the 48 individuals was determined by running a continuous series of K = 1–10 with 5000 burn-in iterations and 50,000 MCMC repetitions in Structure. The calculation of the averaged likelihood at each K [In Pr (X|K) or In (Kn)] was performed in Structure Harvester [45]. For the final K analysis, a burn-in of 50,000 with a run length of 500,000 MCMC replications and 20 independent runs were conducted. Lastly, a neighbor-joining tree was constructed based on the Nei’s genetic distances using the “aboot” function in R package poppr [46] with the “nj” parameter and 500 bootstrapping replicates. The resulting tree was plotted using the R package ggtree [47].

## 3. Results and Discussion

### 3.1. Genetic Basis of Insecticide Resistance

Insecticide resistance is a growing challenge in potato production, and our research provides a foundation for understanding the genetics of insecticide resistance in potato psyllid. In this study, the potato psyllid population that was susceptible to imidacloprid and thiamethoxam [14] allowed us to characterize genotypes and alleles that were significantly different between this susceptible population and the neonicotinoid-resistant populations. We were able to identify a list of genes associated with insect gene regulation, fertility, and development, even though many of the loci containing the strongly segregated SNPs were not annotated (68.4%), as it is likely that they are located on non-coding or intergenic sections of the potato psyllid genome, or that they are unique to potato psyllids.

We generated 288 million pair-end reads with an average of 5.9 M reads per sample. As no reference genome was available for *B. cockerelli*, a de novo assembly was constructed using Stacks. The total ddRAD data set included 456,481 unique loci. We retained a total of 11,471 loci and 12,681 SNPs after filtering loci that did not pass sample/population constraints, and all these loci passed the HWE test (*p*-value < 0.01). Further filtering was also used to select only one SNP at each locus (the first SNP per locus) that generated 4294 SNPs.

**Figure 1:**
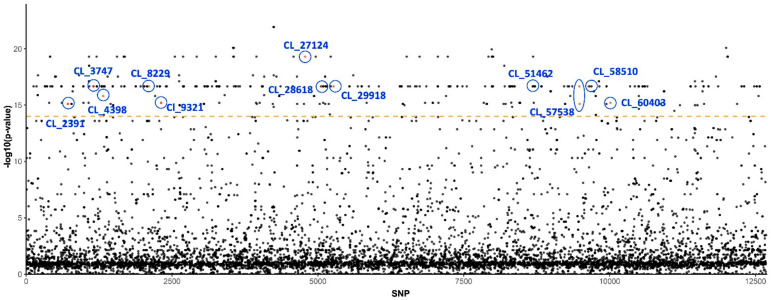
**SNPs and *p*-values from association test with insecticide resistance.** The SNP index on y-axis is sorted by locus names and where they are located. Highlighted loci that host strongly segregated SNPs and their annotation are also presented in Table 2. Orange dashed line indicates the *p*-value cutoff (1 × 10^−14^) for association test.

Based on association test results, we detected 429 SNPs out of 12,681 on 215 unique loci that were strongly segregated between the susceptible and insecticide-resistant populations (Supplementary Table S1, Figure 1). Approximately one-third (68/215) of the unique loci were found to be homologous to known genes or sequences in the annotation database when the E-value cutoff was set as 1 × 10^−4^ (Supplementary Table S2). Notably, a number of these genes have been identified as being associated with insecticide resistance, insect gene control, fertility, and development. Furthermore, a few loci were found to be homologous to genes from microbes that are endosymbiont of or transmitted by potato psyllids, such as genes from *Wolbachia* and Lso (Table 2 and Figure 1).

Within insecticide resistance loci, we found the locus CL_8229 to be homologous of the Paramoysin (*prm*) gene, which was reported to be one of the two key loci involved in imidacloprid resistance in populations of *Drosophila melanogaster* based on a large-scale genome-wide association study (GWAS) [38]. The *prm* encodes a protein that constitutes the structure of muscle filament, and mutation of the *prm* would impact the indirect flight muscle and power generation in *D. melanogaster* [48]. In addition, locus CL_28618 was found to be homologous to wheat stem sawfly, *Cephus cinctus* inositol 1,4,5-trisphosphate receptor (IP3R), a family of the Ca^2+^ release channel protein involved in increasing the cytoplasmic free calcium concentration. Guo et al. [37] reported that silencing of *ip3r* in a silverleaf whitefly, *Bemisia tabaci*, decreased the susceptibility of adult *B. tabaci* to cyantraniliprole, a diamide pesticide that is widely used to manage sucking insect pests. The last locus within this group, CL_51462, is homologous to the *non-lysosomal glucosylceramidase-like* gene. Although the function of glucosylceramidase in insects is not well characterized, it was also reported to be differentially expressed in the abdomen of insecticide-resistant strains of mosquito, *Anopheles gambiae*, a vector of malaria [36].

Within loci related to gene regulation, DNA repair, and innate immunity, we found interesting homologous, including the locus CL_3747, which is homologous to DDB1- and CUL4-associated factor 5 in Asian citrus psyllid, *Diaphorina citri* (Table 2). This locus is involved in gene regulation, DNA repair, and innate immunity, and it is reported to be involved in DNA damage response [49]. Another notable locus, CL_4398, is homologous to *D. citri* histone deacetylase 11. This gene has been proven to be a crucial regulator in insect larval development [50].

Moreover, several loci that differed between the susceptible and resistant populations were related to the innate immunity of insects. For example, CL_2391 is homologous to spaetzle 4-like in *D. citri*, a ligand that responds to the bacterial or fungal infection by binding Toll receptors to induce the secretion of antimicrobial peptides [51,52]. Another locus in this class, CL_9321, is homologous to a pea aphid (*Acyrthosiphon pisum*) CD109 antigen, which is induced by ecdysone signaling and involved in the cellular immunity of insects [53]. It is noteworthy that multiple studies have linked insect immunity pathways to insecticide resistance [54,55,56]. For example, in a recent study characterizing gene expression of *Bt*-resistant and *Bt*-susceptible bollworm, *Helicoverpa zea*, several immunity genes were found to be differentially expressed between resistant and susceptible populations, including two CD109 antigen genes [55].

In addition to insect DNA, we discovered three contigs (including the previously mentioned *Wolbachia* homolog) that presumably originated from the microbiome of potato psyllids (Table 2). Two contigs, CL_27124 and CL_57510, are homologous to Lso, the bacterial pathogen transmitted by potato psyllids. It is unknown whether the plant pathogen that psyllids transmit has any impact on insecticide resistance.

Insecticide resistance has been a prevailing challenge to sustainable pest management and has been the focus of research for decades. Two major mechanisms are thought to result in insecticide resistance: (1) insecticide targeted site gene mutations, which result in target insensitivity [9,12,13,33,34], and (2) increases in detoxification gene products, also known as metabolic resistance [11,13,35]. In the case of target site insensitivity, mutations occur at the active sites of genes that encode proteins that are the targets of insecticides due to long-term insecticide selection. Insecticide resistance can also originate from the target organism’s increased ability to detoxify the pesticide’s active ingredient. Usually, detoxifying enzymes comprise three main superstructures: cytochrome P450 (CYP), glutathione S-transferases (GST), and carboxylesterases (COE). Among these, P450s tend to play a key role in interactions between four plants and insects and have been studied intensively [57,58,59,60]. Several studies employed bioassays that target these genes, and they are informative in detecting the prevalence of insecticide resistance genes [61,62,63].

Beyond these few well-known targets, different insect species might have evolved distinct genes and molecular mechanisms of insecticide resistance [64], and insecticide resistance is often a complex and polygenetic phenotype. Therefore, examining genotypes of insecticide-resistant populations, lines, and individuals offers a different perspective on the origin, expansion, and dynamics of insecticide resistance-related genes or alleles. In a study that used the ddRAD approach, Yang et al. [65] identified several candidate loci strongly associated with population-level resistance to synthetic pyrethroids and organophosphates in a phytophagous mite, *Halotydeus destructor*, a major pest of pastures and crops in Australia. These candidate loci were located in the genomic regions that code for transmembrane transport and signaling proteins, with a few other genes related to pyrethroid resistance. In another study in which whole genome sequencing was conducted on multiple field-collected fruit fly, *D. melanogaster*, two major loci appeared to be related to imidacloprid resistance [38]. One locus was in the coding region of *prm* gene, and another locus was 60 k upstream of the coding region of the *Nicotinic-Acetylcholine Receptor Alpha 3* gene. These studies illustrate the distinct genetic mechanisms of insecticide resistance in different arthropod species.

In our study, we found a strongly segregated SNP located on contigs that is homologous to the *prm* gene. However, we did not identity SNPs that are on contigs that are homologous to previously reported genes involved in insecticide resistance, such as P450, sodium channel gene, *gst*, or *coe*. We speculate that this may be due to the fact that in ddRAD library preparation, only DNA fragments flanking the restriction enzyme cutting sites are included in the DNA library, resulting in just a portion of the genome being sequenced. Furthermore, the lack of a reference genome makes annotating these short de novo assembled tags/loci challenging. We anticipate that the annotation of SNPs will improve with the availability of the reference genome. In the future, examining the transcriptomics will be informative to test whether these SNPs impact the gene expression level of detoxification-related gene products.

We have found multiple variants that are strongly segregated between insecticide susceptible and resistant populations; these variants and related genes could provide a candidate list for further testing. Insecticide resistance is recognized as a complex, polygenic, and quantitative trait, according to growing knowledge and studies on the mechanism and genetic basis of insecticide resistance [9,38]. Thus, the outcomes of our work suggest taking interdisciplinary approaches that include the sampling of natural populations that exhibit a varying degree of resistance, whole genome sequencing, and functional tests to understand this complex trait.

### 3.2. Population Genetic Structure

**Figure 2:**
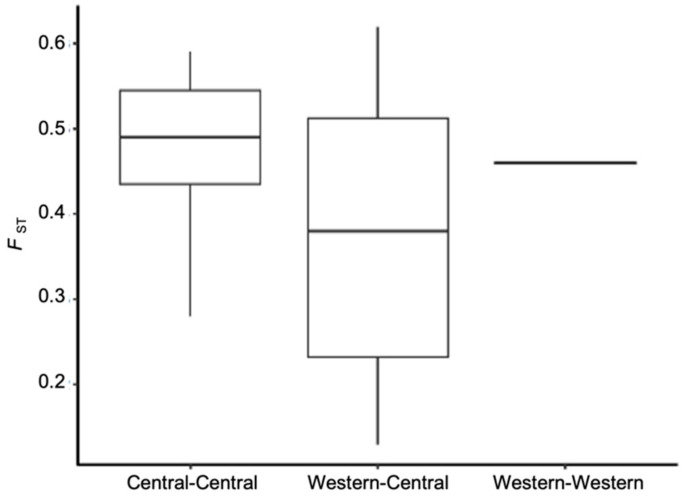
Pairwise *F*_ST_ of psyllid populations separated by whether the pair were different haplotypes or the same haplotype. Note, only two populations were designated as Western haplotype in our study.

The percentage of polymorphic loci and observed and expected heterozygosity (Ho and He respectively) for all loci were estimated (Table 3). The TX Weslaco potato psyllid population was polymorphic at the highest percentage of loci (10%), while all other populations were polymorphic at 6 to 9% of loci. The average observed heterozygosity (Ho) among all populations ranged from 1.6 × 10^−4^ to 2.5 × 10^−4^, and the highest was observed in the TX Weslaco population. To examine variance in allele frequencies among populations and their degree of differentiation, we also measured the fixation index (*F*_ST_) using the data set containing 4294 independent SNPs. The mean *F*_ST_ for all pairwise comparisons was 0.44. The TX Panhandle was, on average, the most differentiated from all other populations (mean *F*_ST_ = 0.52), and Western-2 was the least differentiated population (mean *F*_ST_ = 0.31) (Table S1). Surprisingly, pairwise *F*_ST_ of populations of Central haplotype were not significantly different from pairwise *F*_ST_ of populations that belonged to different haplotypes, e.g., Central and Western (*t*-test, *p*-value = 0.095, Figure 2, Table S1).

**Figure 3:**
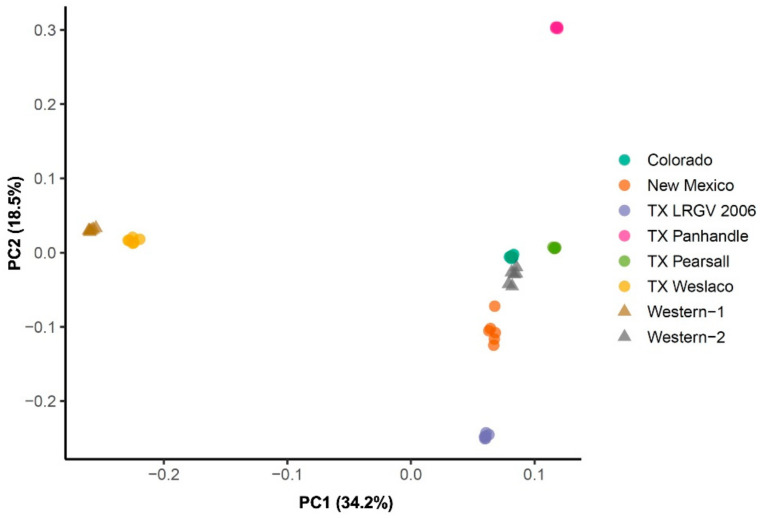
Principal component analysis of eight psyllid populations. PCA implemented in the PLINK using 4294 neutral SNPs marker set.

Further, the principal component 1 (PC1), which explained over 30% of the overall variation (Figure 3), separated the TX Weslaco and Western-1 cluster from the rest of the six populations. At PC2, TX LRGV 2006 and TX Panhandle diverged from the rest of the four populations, i.e., TX Pearsall, Colorado, Western-2, and New Mexico. The populations from New Mexico, Colorado, Western-2, and TX Pearsall grouped together. Similar to the *F*_ST_ results, we did not find that populations from the same haplotypes clustered together, e.g., Western-1 and Western-2 did not cluster together, and TX Weslaco was separated from the other Central haplotype populations.

**Figure 4:**
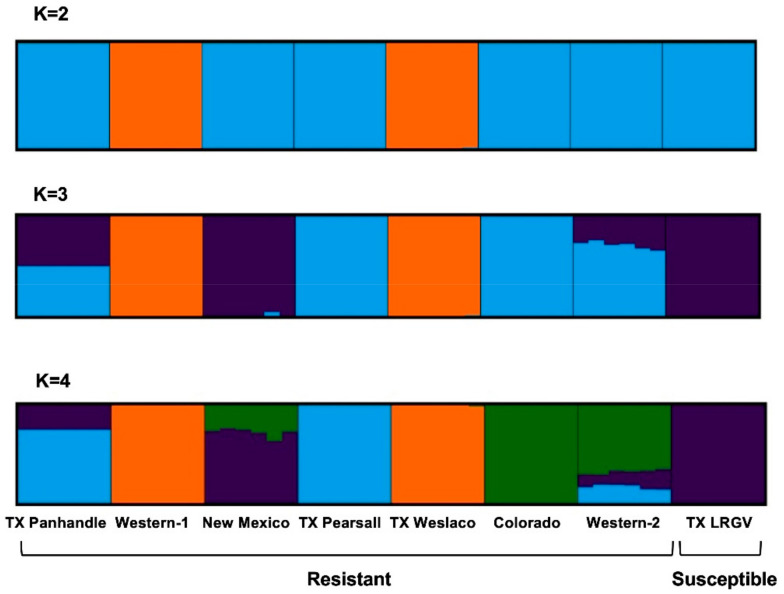
Cluster assignment of the individuals revealed by Structure. Each individual is represented by a single column divided into K genetic clusters. K indicates the number of clusters that maximized the probability of the model. The color proportions of each bar correspond to individuals’ estimated membership fractions of each of the clusters.

Moreover, Structure analysis indicated the most likely number of genetic clusters to be four (K = 4), based on the agreement between the ΔK method and estimators of the structure selector. At K = 2, Western-1 and TX Weslaco were placed in one cluster (Figure 4), and the rest of the six populations were placed in a different cluster. At K = 3, New Mexico and TX LRGV formed a new cluster that separated them from Western-1 and TX Weslaco. TX Panhandle and Western-2 seemed to be admixed between the TX Pearsall‒Colorado cluster and the New Mexico‒TX LRGV cluster. At K = 4, the Colorado samples separated and formed their own cluster, and Western-2 seemed to be most similar to Colorado while sharing some genetic similarities to TX Pearsall and TX LRGV. This pattern was consistent with the PCA outcomes. The overall pattern of the genetic structure indicated that populations of the same haplotype did not cluster together.

**Figure 5:**
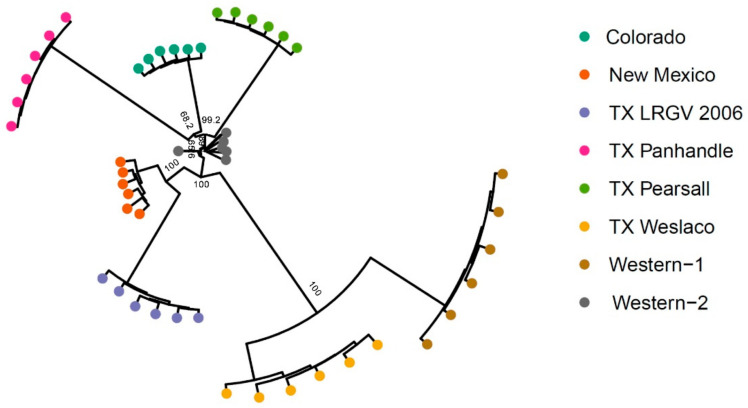
Neighbor-joining tree of 48 samples from eight populations. Five hundred bootstrapping resampling was conducted to obtain the support values indicated at the branch. Support values are not labeled for the end branch because the eight samples within a population are rather similar, given they were pooled samples.

To further examine the genetic structure of psyllid populations, we used the 4294 neutral SNPs data set to conduct a phylogenetic analysis based on Nei’s distance. Similar to results in PCA and Structure, TX Weslaco and Western-1 were found to be closely related. The New Mexico and TX LRGV 2006 populations clustered together, and three TX Panhandle, Colorado, and TX Pearsall populations were found to be closely related (Figure 5). All Western-2 samples seemed to form a tight cluster with the exception of one sample. The neighbor-joining tree revealed a pattern similar to PCA, Structure, and *F*_ST_, suggesting that haplotype does not appear to be a good indication of genetic structure.

Based on genetic structure results, the susceptible population (TX LRGV) was found to be somewhat related to the population of New Mexico; however, the ***F*_ST_** between the two is moderate (Table S1). We found no genetic structure linkage to haplotypes, as the two Western haplotype psyllid populations did not cluster together using any of the three methods tested, including PCA, Structure, and neighbor-joining tree. Similarly, the pairwise *F*_ST_ value of the populations that originated from different haplotypes was not different from the *F*_ST_ from the same haplotype population pairs. This suggests that haplotyping is not an informative marker to determine the genetic differentiation of potato psyllids. Similar results were shown in other studies that examined both haplotypes and genotypes of potato psyllids [66,67]. Specifically, Fu et al. [67] employed genome-wide SNP to analyze the genetic structure of potato psyllids and found that a handful of Western haplotype psyllids were genetically more similar to psyllids of the Northwestern haplotype. In potato psyllids, haplotyping is based on nucleotide variation within a 500 bp- stretch of the potato psyllid COI gene [20], which is located in the mitochondrial genome and is inherited maternally. This means that if there is any hybridization among psyllids from different haplotypes, the haplotype of the offspring will only reflect the maternal lineage [67]. More broadly, in the animal kingdom, conflicting phylogenetics and population genetics patterns are common between mitochondrial and nuclear DNA markers [68]. We suggest that future research should focus on including a wider range of molecular markers rather than relying solely on COI haplotyping to provide more thorough information on potato psyllid ancestry and lineage.

## Figures and Tables

**Table 1:** List of potato psyllid colonies included in this study.

Pop ID	Haplotype	Original Collection Location	Collection Time	Resistance to Insecticide
Colorado	Central	Wray, CO	2015	Resistant
New Mexico	Central	Farmington, NM	2015	Resistant
TX LRGV	Central	Weslaco, TX	2006	Susceptible
TX Panhandle	Central	Dalhart, TX	2016	Resistant
TX Pearsall	Central	Pearsall, TX	2014	Resistant
TX Weslaco	Central	Weslaco, TX	2015	Resistant
Western-1	Western	Unknown	2013	Resistant
Western-2	Western	Unknown	2015	Resistant

**Table 2:** A subset of loci that host strongly segregated SNPs between insecticide susceptible and resistant psyllid populations.

SeqName	Description	e-Value	Number of Variant Sites
CL_2391	*Diaphorina citri* protein spaetzle 4-like	1.98 × 10^−23^	2
CL_3747	*Diaphorina citri* DDB1- and CUL4-associated factor 5	1.55 × 10^−5^	4
CL_4398	*Diaphorina citri* histone deacetylase 11	1.57 × 10^−4^	1
CL_8229	*Diaphorina citri* paramyosin	1.36 × 10^−11^	1
CL_9321	*Acyrthosiphon pisum* CD109 antigen	9.81 × 10^−20^	2
CL_28618	*Cephus cinctus* inositol 1,4,5-trisphosphate receptor	9.22 × 10^−14^	1
CL_29918	*Diaphorina citri* neurogenic locus Notch protein	3.17 × 10^−39^	1
CL_51462	*Diaphorina citri* non-lysosomal glucosylceramidase-like	4.66 × 10^−12^	1
CL_60403	*Diaphorina citri* angiotensin-converting enzyme 2-like	7.22 × 10−9	1
CL_27124	*Candidatus* Liberibacter solanacearum CLso-ZC1	1.09 × 10^−37^	1
CL_57538	*Wolbachia* endosymbiont of Culex molestus DNA methylase-like protein	1.04 × 10^−45^	2
CL_58510	Candidatus Liberibacter solanacearum CLso-ZC1	4.30 × 10^−50^	1

**Table 3:** Summary of genetics statistics calculated by the Stacks. Private refers to number of variable sites unique to each population; %Polymorphic_Loci refers to percentage of polymorphic loci, Obs_He and Exp_Het refer to average observed and expected heterozygosity per locus, respectively.

Pop ID	Private	%Polymorphic_Loci	Obs_Het	Exp_Het
Colorado	118	2.78 × 10^−2^	1.8 × 10^−4^	1.1 × 10^−4^
New Mexico	72	3.12 × 10^−2^	1.9 × 10^−4^	1.2 × 10^−4^
TX LRGV	161	2.81 × 10^−2^	1.7 × 10^−4^	1.1 × 10^−4^
TX Panhandle	356	2.35 × 10^−2^	1.6 × 10^−4^	9.0 × 10^−5^
TX Pearsall	235	2.55 × 10^−2^	1.7 × 10^−4^	1.0 × 10^−4^
TX Weslaco	167	3.60 × 10^−2^	2.5 × 10^−4^	1.5 × 10^−4^
Western-1	249	3.32 × 10^−2^	2.3 × 10^−4^	1.4 × 10^−4^
Western-2	29	3.15 × 10^−2^	1.6 × 10^−4^	1.1 × 10^−4^

## Data Availability

All raw sequencing reads have been submitted to the NCBI Sequence Read Archive and are available under BioProject ID: PRJNA773566: https://www.ncbi.nlm.nih.gov/sra/PRJNA773566 (accessed on 19 October 2021).

## Supplementary Materials

The following supporting information can be downloaded at: https://www.mdpi.com/article/10.3390/insects13030257/s1, Table S1: pairwise comparison of genetic (*F_ST_*) among eight populations of potato psyllid based on 4294 independent loci, Table S2: significant SNPs for association test.
