# Supplementary material for: ddRAD Sequencing Identifies Pesticide Resistance-Related Loci and Reveals New Insights into Genetic Structure of Bactericera cockerelli as a Plant Pathogen Vector
Source: Insects. 2022 Mar 4;13(3):257. doi: 10.3390/insects13030257 (PMC8950205; doi:10.3390/insects13030257)
Supplement: Supplementary file 1 [file insects-13-00257-s001.zip › Table S1.pdf]

Table S1. Pairwise comparison of genetic (F_{ST}) among eight populations of potato psyllid based on 4294 independent loci.

	Colorado	New Mexico	TX LRGV	TX Panhandle	TX Pearsall	TX Weslaco	Western 1	Western 2
**Colorado**	0.00							
**New Mexico**	0.33	0.00						
**TX LRGV**	0.49	0.28	0.00					
**TX Panhandle**	0.49	0.45	0.59	0.00				
**TX Pearsall**	0.42	0.40	0.54	0.54	0.00			
**TX Weslaco**	0.50	0.47	0.55	0.59	0.56	0.00		
**Western 1**	0.55	0.50	0.59	0.62	0.50	0.13	0.00	
**Western 2**	0.21	0.19	0.32	0.36	0.24	0.40	0.46	0.00
